# Addressing racial and ethnic disparities in AACR project GENIE

**DOI:** 10.1038/s41698-023-00425-5

**Published:** 2023-08-31

**Authors:** Shawn M. Sweeney, Jessica A. Lavery, Hannah E. Fuchs, Jocelyn A. Lee, Samantha Brown, Katherine S. Panageas, Charles L. Sawyers, Philippe L. Bedard

**Affiliations:** 1 AACR Project GENIE, American Association for Cancer Research, Philadelphia, PA USA; 2 Department of Epidemiology and Biostatistics, Memorial Sloan Kettering Cancer Center, New York, NY USA; 3 Human Oncology and Pathogenesis Program, Memorial Sloan Kettering Cancer Center, New York, NY USA; 4 Howard Hughes Medical Institute, Chevy Chase, MD USA; 5 Division of Medical Oncology & Hematology, University Health Network - Princess Margaret Cancer Centre, Toronto, ON Canada

**Keywords:** Cancer genomics, Epidemiology

**arising from** Cheung et al. *npj Precision Oncology* 10.1038/s41698-023-00351-6 (2023)

AACR Project GENIE is an open source, international, pan-cancer registry of real-world clinico-genomic data built by data sharing among a network of academic tertiary referral centers. We write in response to a recent report by Cheung and colleagues^1^ who claim, based on an analysis of the distribution of race and ethnicity in GENIE and benchmarking those distributions to 2017 U.S. cancer incidences using CDC WONDER (http://wonder.cdc.gov/cancer-v2017.html), that “GENIE is not sufficiently powered to detect small yet potentially clinically meaningful differences between white and non-white patients in even the most common cancer types.”

We disagree with Cheung et al.’s emphasis on powering comparisons for a Cohen’s h of less than 0.2. As per Cohen’s guidelines^2^, h of 0.2 is the benchmark for a small effect size. Using this definition, comparisons of Black, Asian, and Hispanic primary tumor samples versus white samples from three of the top five most common cancers are adequately powered to achieve this small effect size. This benchmark is also met for comparisons in the metastatic setting of white versus: Black and Asian non-small cell lung cancer samples; Black, Asian and Hispanic breast cancer samples; and Black and Hispanic colorectal cancer samples.

Cheung et al. used the v9.1 public release (January 2021) for their analysis. GENIE has updated public releases every 6 months, which include both new patients and samples. Using the most recent release from January 2023 (v13.0-public)^3^, several previously underpowered comparisons with white patient samples (Black primary prostate cancer samples, Hispanic primary pancreatic cancer and metastatic non-small cell lung cancer, and Asian metastatic colorectal cancer samples) are now sufficiently powered for Cohen’s h of 0.2. As GENIE continues to collect data and as new centers with currently underrepresented patient populations join the consortium, each release will include more patients across all race and ethnicity groups. This will allow for smaller effect sizes to be detected, as well as for additional comparisons to be adequately powered.

We agree with Cheung and colleagues on the many challenges to collecting and analyzing self-reported race and ethnicity data. AACR Project GENIE uses the standards established by the North American Association of Central Cancer Registries (NAACR) whenever possible to define specific data elements, including self-reported race and ethnicity (https://www.synapse.org/#!Synapse:syn50678640). Collection of these data at international GENIE institutions is further complicated by varying European Member State laws^4^. Given the complexities and missingness in self-reported race and ethnicity data, GENIE is currently undertaking an infrastructure build to impute genetic ancestry from off-target Next Generation Sequencing (NGS) panel reads^5^.

Clinical research datasets have inherent biases that may limit their generalizability. It is important to consider these limitations when evaluating the appropriateness of a dataset for its intended use^6^. Inclusion in the GENIE Registry requires that a patient’s tumor undergoes NGS testing. As such, the data reflect patient populations and practice patterns at participating institutions, which may not represent the broader population of patients that are diagnosed and treated for cancer^7^. Biobanking studies demonstrate participation bias can lead to false positive inferences about genetic associations and phenotype^8,9^.

The GENIE consortium recently underwent an open call to expand the consortium by adding institutions with clinical and genomic data from cancer patients consistent with the national average of minority and underrepresented patients treated for cancer or who are from rural populations^10,11^. Four institutions were selected and are currently being onboarded, with the first release incorporating data from these patients anticipated in January 2024. The GENIE Consortium is also undertaking several parallel pathways to connect select patient- and area-level social determinants of health variables that should allow for a more comprehensive evaluation of factors that influence variation in outcomes.

The members of the AACR Project GENIE Consortium fully believe in the need for high-quality real-world clinico-genomic data, and agree with Cheung et al. on the importance of racial and ethnic representation in such databases. GENIE and its partnering institutions will continue to adapt in conjunction with changes to practice and health policy so that clinico-genomic data can be captured for as broad and representative a population of patients as is feasible. We look forward to continuing to serve the research community for years to come by providing a publicly available source of high-quality clinico-genomic data.
